# Vorinostat synergises with capecitabine through upregulation of thymidine phosphorylase

**DOI:** 10.1038/sj.bjc.6605969

**Published:** 2010-11-02

**Authors:** E Di Gennaro, G Piro, M I Chianese, R Franco, A Di Cintio, T Moccia, A Luciano, I de Ruggiero, F Bruzzese, A Avallone, C Arra, A Budillon

**Affiliations:** 1 Experimental Pharmacology Unit, Department of Research, Istituto Nazionale Tumori, National Cancer Institute Fondazione G, Via M Semmola, Pascale, Napoli 80131, Italy; 2 Centro di Ricerche Oncologiche di Mercogliano (CROM), Mercogliano (AV) 83013, Italy; 3 Pathology Unit, National Cancer Institute Fondazione G, Pascale, Napoli 80131, Italy; 4 Experimental Animal Unit, National Cancer Institute Fondazione G, Pascale, Napoli 80131, Italy; 5 Gastrointestinal Medical Oncology Unit, National Cancer Institute Fondazione G, Pascale, Napoli 80131, Italy

**Keywords:** HDAC inhibitor, vorinostat, thymidine phosphorylase, thymidylate synthase, colon cancer, capecitabine

## Abstract

**Background:**

Potentiation of anticancer activity of capecitabine is required to improve its therapeutic index. In colorectal cancer (CRC) cells, we evaluated whether the histone deacetylase-inhibitor vorinostat may induce synergistic antitumour effects in combination with capecitabine by modulating the expression of thymidine phosphorylase (TP), a key enzyme in the conversion of capecitabine to 5-florouracil (5-FU), and thymidylate synthase (TS), the target of 5-FU.

**Methods:**

Expression of TP and TS was measured by real-time PCR, western blotting and immunohistochemistry. Knockdown of TP was performed by specific small interfering RNA. Antitumour activity of vorinostat was assessed *in vitro* in combination with the capecitabine active metabolite deoxy-5-fluorouridine (5′-DFUR) according to the Chou and Talay method and by evaluating apoptosis as well as in xenografts-bearing nude mice in combination with capecitabine.

**Results:**

Vorinostat induced both *in vitro* and *in vivo* upregulation of TP as well as downregulation of TS in cancer cells, but not in *ex vivo* treated peripheral blood lymphocytes. Combined treatment with vorinostat and 5′-DFUR resulted in a synergistic antiproliferative effect and increased apoptotic cell death *in vitro*. This latter effect was impaired in cells where TP was knocked. *In vivo*, vorinostat plus capecitabine potently inhibited tumour growth, increased apoptosis and prolonged survival compared with control or single-agent treatments.

**Conclusions:**

Overall, this study suggests that the combination of vorinostat and capecitabine is an innovative antitumour strategy and warrants further clinical evaluation for the treatment of CRC.

Although higher response rates have been achieved using the latest poly-chemotherapy regimens with fluoropyrimidines (5-florouracil (5-FU) and capecitabine) representing the backbone of therapies including agents such as oxaliplatin or irinotecan and/or new molecular targeted drugs such as cetuximab or bevacizumab, colorectal cancer (CRC) remains the second leading cause of cancer death in the Western countries. Resistance to chemotherapy and multi-step carcinogenesis that limits the efficacy of targeted compounds are probably the leading causes of the limited activity of anticancer strategies in CRC.

Histone deacetylase inhibitors (HDAC-Is) represent a new class of anticancer agents that modulate the expression of cell cycle regulation, survival and differentiation genes by enhancing histone acetylation, thus affecting multiple pathways in tumour cells with minimal effects on normal tissues (Budillon *et al*, 2005, 2007; Marks, 2007; Lane and Chabner, 2009). Several HDAC-Is exhibited antitumour effects in preclinical models (Budillon *et al*, 2007), and some of them are in advanced clinical development either as single agents or in combination with conventional chemotherapeutics or targeted agents (Budillon *et al*, 2007; Lane and Chabner, 2009). Histone deacetylase inhibitors act very selectively to alter the transcription of 2–5% of expressed genes (Budillon *et al*, 2007) by mechanisms that cannot be solely attributed to the level of histone acetylation (Johnstone and Licht, 2003). Acetylation of other proteins such as p53, *α*-tubulin and heat shock protein 90, have also been described (Johnstone and Licht, 2003). Among the most promising HDAC-Is, vorinostat (suberoylanilide hydroxamic acid) has shown significant preclinical and clinical antitumour activity in both haematological malignancies and solid tumours and represents the first HDAC-I to be approved by the Food and Drug Administration for the treatment of cutaneous T-cell lymphoma in patients with progressive, persistent or recurrent disease during or following two systemic therapies (Marks, 2007).

Several studies have shown that the expression of the enzyme thymidylate synthase (TS) can be regulated by HDAC-Is (Glaser *et al*, 2003; Lee *et al*, 2006; Tumber *et al*, 2007; Di Gennaro *et al*, 2009; Fazzone *et al*, 2009). Thymidylate synthase is an essential enzyme for the *de novo* synthesis of thymidilate (dTMP) and subsequently DNA synthesis and is a critical target for 5-FU Ackland *et al*, 2002). High levels of TS expression have been correlated with poorer overall patient survival in several tumours and resistance to 5-FU (Ackland *et al*, 2002). We have recently demonstrated that vorinostat induces synergistic antitumour activity in CRC cell lines in combination with either 5-FU or raltitrexed, a selective quinazoline antifolate TS inhibitor, and sensitises 5-FU-resistant CRC cells to 5-FU. We have also shown that down-modulation of TS protein induced by vorinostat within 24 h represented a key factor in enhancing the effects of both drugs in sensitive and resistant tumour cells (Di Gennaro *et al*, 2009).

Although the antineoplastic activity of 5-FU could be improved by continuous infusion or biochemical modulation with leucovorin, the inconvenience and morbidity associated with long-term central venous catheters suggested the need of alternative regimens. Capecitabine is an orally administered inactive prodrug that is converted into 5-FU by a three-step process *in situ*. The key step, the conversion of deoxy-5-fluorouridine (5′-DFUR) into active 5-FU is catalysed by thymidine phosphorylase (TP), which is expressed at elevated levels by the liver and many tumours, allowing capecitabine to be specifically targeted to the site of the cancer, leading to relatively high local concentrations of 5-FU in tumour cells (Bollag and Hartmann, 1980; Walko and Lindley, 2005). Cytotoxicity of 5-FU mainly results from incorporation of the drug into replicating RNA after the conversion to FUTP and thymidine depletion following TS inhibition induced by FdUMP. Thymidine phosphorylase can convert 5-FU to FdUrd by adding deoxyribose-1-P donors, which are readily phosphorylated by thymidine kinase to FdUMP (de Bruin *et al*, 2003). Consequently, TP represents the rate-limiting enzyme in the activation of 5′-DFUR and capecitabine, suggesting that increasing TP expression might enhance sensitivity of tumour cells to these prodrugs. In this regard, preclinical and clinical studies showed that overexpression of TP by direct transfection of human TP cDNA into cancer cells or its induction by cytokines, chemotherapeutics such as cyclophosphamide and taxanes or by irradiation resulted in increased sensitivity to 5′-DFUR or capecitabine (Haraguchi *et al*, 1993; Patterson *et al*, 1995; Kato *et al*, 1997; de Bruin *et al*, 2003; Toi *et al*, 2005). Conversely, suppression of TP expression by specific small interfering RNA (siRNA) in cancer cells and low or deficient intratumoural TP activity impaired 5-FU therapeutic efficacy in preclinical studies and in cancer patients, respectively (Salonga *et al*, 2000; de Bruin *et al*, 2003; Thanasai *et al*, 2010). These data provided additional evidence supporting the importance of TP in fluoropyrimidine sensitivity.

Clinical trials with single-agent capecitabine showed improved tolerability and comparable efficacy to intravenous 5-FU/leucovorin therapy in patients with metastatic CRC, leading to approval of capecitabine for use as first-line therapy in this disease (Walko and Lindley, 2005; Schmoll and Arnold, 2006; Cassidy *et al*, 2008).

These considerations have compelled us to study whether vorinostat and capecitabine used in combination could have synergistic antitumour effects. In the present study, we provided evidence that by downregulating TS and upregulating TP mRNA and protein expression, vorinostat demonstrated synergistic antitumour effects both *in vitro* and *in vivo* in combination with 5′-DFUR and capecitabine, respectively.

## Materials and Methods

### Materials

Vorinostat was provided by Merck and Co., Inc. (Rahway, NJ, USA). Stock solutions were prepared in DMSO and diluted to appropriate concentrations in culture medium before addition to the cells. MS-275, trichostatin A (TSA), valproic acid (VPA) were obtained from Alexis Biochemicals (San Diego, CA, USA), LBH589 was provided by Novartis Pharmaceuticals (East Hanover, NJ, USA), tubacin and niltubacin were provided by Dr SL Schreiber and Dr Ralph Mazitschek (Haggarty *et al*, 2003). 5-Fluorouracil (5-FU) was supplied by Teva Pharmaceutical Industries Ltd. (Netanya, Israel), folinic acid (FA) was obtained from Wyeth Pharma GmbH (Münster, Germany) and 5′-deoxy-5-fluorouridine (5′-DFUR) and capecitabine (Xeloda) were provided by F Hoffmann-La Roche Ltd. (Basel, Switzerland). All media, serum, antibiotics and glutamine were from Cambrex Bio Science (Verviers, Belgium).

### Cell culture and cell proliferation assay

LoVo, SW620, HT29 and LS174T cell lines were all from American Type Culture Collections (Rockville, MD, USA); 5-FU-resistant HT29 cell lines (HT29FU) have been described before (Di Gennaro *et al*, 2009). The LoVo cell line was grown in RPMI 1640 medium, whereas HT29, HT29FU, SW620 and LS174T were grown in Dulbecco's modified Eagle's medium. Both media were supplemented with 10% heat-inactivated foetal bovine serum, 50 U ml^−1^ of penicillin, 500*μ*g ml^−1^ of streptomycin, and 4 mmol l^−1^ of glutamine in a humidified atmosphere of 95% air and 5% CO_2_ at 37 °C.

Cell survival/proliferation was measured in quadruplicate in 96-well plates by a spectrophotometric dye incorporation assay using sulforhodamine B (ICN Biomedicals, Irvine, CA, USA) after 96 h from treatment, as described before (Di Gennaro *et al*, 2009).

Carboxylesterase activity is required as the first metabolic step in the activation of capecitabine, but this enzyme has low-level expression in most cancer cell lines. Thus, all *in vitro* studies in cancer cells were performed with capecitabine-metabolite 5′-DFUR, which requires the presence of TP to be converted into the active 5-FU drug. Because FA was tested at 10 *μ*M and did not exhibit any effect on cell proliferation when given alone, 5-FU/FA was considered a single drug. Peripheral blood lymphocytes (PBLs) were obtained from a healthy donor by gradient separation with Lymphocyte separation medium (BioWhittaker, Walkersville, MD, USA) and grown for 24 h in RPMI. Floating lymphocytes were collected and treated with the indicated concentration of vorinostat.

### *In vitro* drugs combination studies

Drug combination studies were based on concentration-effect curves generated as a plot of the fraction of unaffected (surviving) cells *vs* drug concentration (Chou and Talalay, 1984; Chou *et al*, 1994). Serial dilutions of equipotent doses of the two agents in combination (vorinostat and 5′-DFUR) were tested. Synergism, additivity or antagonism was quantified by determining the combination index (CI) calculated by the Chou–Talalay equation, as described elsewhere (Magne *et al*, 2002; Bruzzese *et al*, 2006, 2009; Avallone *et al*, 2007; Di Gennaro *et al*, 2009). A CI <0.9, CI=0.9–1.2 and CI: >1.2 indicated synergistic, additive or antagonistic effect, respectively (Magne *et al*, 2002; Di Gennaro *et al*, 2009). Dose reduction index (DRI) represents the measure of how much the dose of each drug in a synergistic combination may be reduced at a given effect level compared with the doses of each drug alone (Chou and Talalay, 1984).

### Protein extraction and western blotting

Cells grown and treated as indicated were collected, lysed and separated on SDS polyacrilamide gel electrophoresis, as described elsewhere (Bruzzese *et al*, 2006; Di Gennaro *et al*, 2009). After electrophoresis, proteins were transferred to nitrocellulose paper, immunoblotted with specific antibodies (Abs) and probed with the appropriate horseradish peroxidase-linked IgG. Immunoreactive bands were detected by enhanced chemiluminescence (GE Healthcare, Milan, Italy). The following primary Abs were used: thymidylate synthase (TS)-Ab from Rockand Immunochemicals, Inc. (Gilbertsville, PA, USA), acetyl-H3-Ab from upstate (Lake Placid, NY, USA), poly-(ADP-ribose)-polymerase (PARP)-Ab from BD Biosciences (San Jose, CA, USA), *γ*-tubulin-Ab from Santa Cruz Biotechnology (Santa Cruz, CA, USA), platelet-derived endothelial growth factor (TP)-Ab from Acris Antibodies GmbH (Herford, Germany) and GAPDH-Ab, cleaved caspase 3-Ab and BAX-Ab from Cell Signalling Technology (Boston, MA, USA). Densitrometric analysis of western blotting data was performed by NIH Image J software (National Institutes of Health, Bethesda, MD, USA).

### Flow cytometry analysis of apoptosis

Analysis of apoptosis was performed on LoVo cells treated with vorinostat and/or 5′-DFUR, at the indicated concentrations, after 72 h of treatment. Briefly, adherent and floating cells were harvested, fixed in 70% ethanol and stored at −20 °C until analysis. After nuclear DNA staining with propidium iodide, flow cytometry was done by a FACScan flow cytometer (Becton Dickinson, San Jose, CA, USA). Apoptosis was determined by evaluating the percentage of cells with DNA content <2N, PI fluorescence was collected as FL3 (Log scale) by the CellFIT software (Becton Dickinson, San Jose, CA, USA) and the data were acquired after analysis of at least 20 000 events.

### Reverse transcription-PCR (RT–PCR) and real-time PCR

RNA was isolated using the Trizol reagent as indicated by the manufacturer's instructions (Invitrogen, Carlsbad, CA, USA). The RT–PCR assay was performed using the High capacity cDNA reverse transcription kit (Applied Biosystems, Foster City, CA, USA).

TS (Hs00926280_m1), TP (TYMP, Hs00157317_m1) and GADD45 (Hs99999173_m1) mRNA expressions were quantified using the ABI Prism 7900 HT Sequence Detection System (Applied Biosystems, Foster City, CA, USA) utilising the 5′-nuclease method with a two-step PCR protocol (95 °C for 10 min, followed by 40 cycles for 15 s and 60 °C for 1 min). Each gene was tested in each cell line in three replicates, and three independent experiments were performed. To quantify the relative changes in gene expression, the −2^ΔΔCT^ method (Livak and Schmittgen, 2001) was used, and reactions were normalised to endogenous *β*-actin gene expression levels (Human ACTB, Applied Biosystems, Foster City, CA, USA).

### Transient knockdown of TP using siRNA

siRNA sequences against human TP were designed and synthesised by Stealth RNAi, oligo ID HSS141896 (Invitrogen). A stealth RNAi Negative control Duplexes (Invitrogen) was used as a control for non-sequence-specific effects.

Thymidine phosphorylase siRNA or control siRNA (33 nM) was transiently transfected into SW620 cells using Lipofectamine 2000 (Invitrogen) according to the manufacturer's instructions. Briefly, siRNA : Lipofectamine mixure complexes were incubated for 20 min at room temperature and then added to cells growing in 100-mm petri dish. After 24 h the complexes were replaced by complete medium and cells were treated as indicated and assayed as reported.

### *In vivo* xenograft assay

Female BALB/c athymic (nu+/nu+) mice that were 5–6 weeks of age and weighed 21–23 g were purchased from Charles River Laboratories (Milan, Italy). The research protocol was approved, and mice were housed and maintained under specific pathogen-free conditions in the Animal Care Facility of the G Pascale National Cancer Institute in accordance with the institutional guidelines of the Italian Ministry of Health Animal Care and Use Committee. Mice, housed five per cage, were supplied with food and water *ad libitum*, maintained under aseptic conditions in a ventilated rack system and were acclimatised for 1 week before being injected with cancer cells. SW620 cells (5 × 10^6^) in 200 *μ*l PBS were injected subcutaneously in the right flank area of the mice. When established tumours were palpable, mice were randomised into four experimental groups (*n*=9). Based on pilot studies (data not shown) and previous reports (Butler *et al*, 2000; Fujimoto-Ouchi *et al*, 2001; Blanquicett *et al*, 2005), mice were treated p.o. with vorinostat (100 mg kg^−1^ melted in DMSO and diluted in PEG) and/or capecitabine (359 mg kg^−1^ in 40 mM citrate buffer (pH 6) containing 5% gum arabic) 5 days/week. Each drug was given in a volume of 100 *μ*l, and mice were treated with vorinostat in the morning and with capecitabine in the afternoon. Mice in the control group were treated with both PEG and citrate buffer/Arabic gum vehicles. Tumour size was measured with a caliper two to three times per week by the modified ellipsoid formula (*π*/6) × *AB*^*2*^, where *A* is the longest and *B* is the shortest perpendicular axis of an assumed ellipsoid corresponding to tumour mass (Bruzzese *et al*, 2006). Body weight was measured three times per week to control for treatment toxicity. Mice were killed by cervical dislocation when evidence of advanced bulky disease was present (established cutoff about 2000 mm^3^). The day of killing was considered the day of death for survival evaluation. Survival analysis was computed by the Kaplan–Meier method. Tumour growth delay (TGD) was determined as %TGD=((T−C)/C) × 100, where *T* and *C* are the mean times in days required to reach the same fixed tumour volume in the treated group and control group, respectively (Bruzzese *et al*, 2006). *In vivo* drug combination studies were evaluated by CalcuSyn (Biosoft, Cambridge, UK). For the calculation of CI, the values of cell kill for a fixed tumour volume were considered (determined by the log cell kill (LCK)). Log cell kill was determined as LCK=(T−C)/(3.3−T_d_), where *T*_d_ represents the mean control group doubling time required to reach a fixed tumour volume, expressed in days, whereas *T* and *C* are the same values as described above (Bruzzese *et al*, 2006).

### Histology and immunohistochemistry (IHC)

SW620 xenografts were collected, fixed in 10% buffered formalin and paraffin-embedded. Five sections from each tissue were stained, one with hematoxylin and eosin stain (H&E), one for terminal deoxynucleotidyl transferase (TdT)-mediated dNTP-labelling (TUNEL) assay and three for IHC. Sections 4 *μ*m-thick were deparaffinised and rehydrated, and the antigen retrieval technique was performed in pH 6.0 buffer in a microwave for 3 min. Hematoxylin and eosin staining was performed using standard histological technique. For IHC, the sections were incubated with the following primary Abs: monoclonal mouse thymidylate synthase-Ab (TS106, Dako, Glostrup, Denmark), monoclonal mouse platelet-derived endothelial growth factor (TP)-Ab (Vision biosystems Novocastra, Newcastle, UK) or monoclonal rabbit acetil-H3-(Lys9/Lys14)-Ab (Cell Signaling Technology, Boston, MA, USA). The sections were then processed with a peroxidase detection system reagent kit (Novocastra, Newcastle, UK). Apoptosis was evaluated by the TUNEL method using the Fragel DNA fragmentation detection kit colorimetric-TdT enzyme by Calbiochem–Merck KgaA (Darmstadt, Germany). Necrotic areas were evaluated as a percentage comparing the total tumour volume to the volume of inner tumoural necrosis and were evaluated for all tumours. The mitotic index was expressed as number of mitotic figures in 10 high-magnification fields. Tumour sections stained for TUNEL, TS, TP or acetil-H3 were scored based on percentage of positive cells. Study slides were examined by a single pathologist (RF), who was blinded to the final pathology interpretation. Images were captured using a light microscope with a 20 × or 40 × objective using standard bright-field illumination. The group means were calculated at *n*=5–7 tumours per group. All data are presented as the average±s.d.

### Statistics

The results of *in vitro* cell proliferation are expressed as the means for at least three independent experiments done in quadruplicates, and the s.d. is indicated.

The results of apoptosis by flow cytometry analysis and real-time PCR were expressed as the means for at least three independent experiments (±s.d.), and the statistical significance of differences was determined by two-sided Student's *t-*test.

Representative results from western blotting and IHC from a single experiment are presented; additional experiments yielded similar results. Statistical analysis of *in vivo* mouse survival was carried out by the *χ*^2^-test. Statistical significance of differences in tumour growth and in IHC results were determined by the one-way ANOVA and Holm–Sidak methods, and a *P*-value of <0.05 was used to indicate statistical significance. All statistical evaluations were done using Sigma Stat software (Systat Software Inc., San Jose, California, USA).

## Results

### Antiproliferative effects of vorinostat and fluoropyrimidines in CRC cell lines

All examined CRC cell lines were equally sensitive to the antiproliferative effect of vorinostat. Two out of five cell lines (HT29, LoVo) were sensitive to all fluoropyrimidines tested (5-FU, 5-FU modulated by FA, 5′-DFUR). SW620 cells were resistant to the capecitabine metabolite 5′-DFUR, whereas two cell lines, LS174T and HT29-FU (a subline selected by continuous exposure of sensitive HT29 cells to step wise increasing concentration of 5-FU; Di Gennaro *et al*, 2009), appeared resistant to all fluoropyrimidines (Table 1).

**Figure 1:**
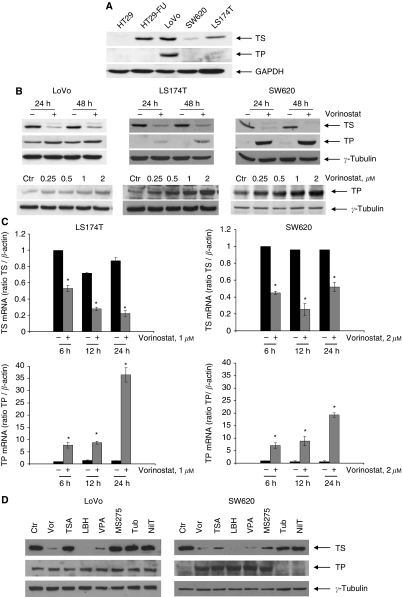
Effect of vorinostat and of other HDAC-Is on TS and TP expression in CRC cells. (**A**) Basal protein expression of TS and TP were analysed by western blotting in the CRC cell lines. GAPDH was used as protein loading control. (**B**) TS and TP proteins expression were determined by western blotting on the indicated CRC cells untreated or treated with vorinostat at concentration corresponding to IC_50_^72 h^ (1 *μ*M LoVo and LS174T cells and 2 *μ*M for SW620 cells) and harvested at indicated times. *γ*-Tubulin was used as protein loading control. (**C**) TS and TP mRNA expression were determined by real-time PCR, as described in ‘Materials and Methods’, on the indicated CRC cells untreated or treated with vorinostat at indicated concentrations and harvested at indicated times. Bars, SD. ^*^P⩽0.005. (**D**) TS and TP proteins expression were determined by western blotting on LoVo and SW620 cells untreated or treated for 24 h with different HDAC-Is at concentration corresponding to IC_50_^72 h^ (LoVo cells: vorinostat 1 *μ*M, TSA 0.02 *μ*M, LBH589 0.34 *μ*M, VPA 2.8 mM, MS275 2.6 *μ*M, tubacin and niltubacin 5 *μ*M; SW620 cells: vorinostat 2 *μ*M, TSA 0.02 *μ*M, LBH589 0.94 *μ*M, VPA 9.2 mM, MS275 2 *μ*M, tubacin and niltubacin 5 *μ*M). *γ*-Tubulin was used as protein loading control.

Apparently, KRAS and p53 status or the basal expression of TS protein, the enzyme target of 5-FU, did not correlate with the sensitivity to fluoropyrimidines and vorinostat. However, undetectable expression of TP protein, the critical enzyme in conversion of 5′-DFUR to the active compound 5-FU, correlates with resistance to 5′-DFUR in three out of four cell lines (Table 1 and Figure 1A).

### Vorinostat and other HDAC-Is modulate TS and TP expression in CRC cell lines

As previously reported by us and other groups, vorinostat induced a marked reduction of TS protein level within 24 h of treatment in LoVo, LS174T and SW620 cell lines. Surprisingly, we observed that TP protein expression was induced by vorinostat in all cell lines after 24 and 48 h, and this effect was dose-dependent and was clearly induced also by low-dose (0.25 *μ*M) vorinostat (Figure 1B). Even in LoVo cells, where the upregulation of TP was less evident, densitometric analysis demonstrated a two-fold increase of the protein expression in cells treated with 0.25 *μ*M of vorinostat, and about a four-fold increase in cells treated with 2 *μ*M of vorinostat.

Furthermore, as shown in Figure 1B and C, vorinostat-induced downregulation of TS, as well as upregulation of TP protein levels, correlates with attenuation and increase of TS and TP mRNA transcript levels, respectively. Specifically, we observed a clear downregulation of TS transcript in LS174T cells after 6 h with a four-fold reduction after 24 h, paralleled by an upregulation of TP transcript that was also evident after 6 h with a 27-fold induction at 24 h. Similar effects were observed in SW620 cells, with a peak of four-fold reduction of TS transcript at 12 h and a peak 30-fold induction of TP transcript at 24 h.

Finally, in order to confirm that our findings were shared by other HDAC-Is, we tested the effects of several inhibitors with different HDACs specificity on both TS and TP expression in LoVo and SW620 cells. As shown in Figure 1D, we demonstrated the induction of TP expression by other pan-HDAC-Is such as LBH589 or TSA and by class I HDAC-Is such as VPA and MS275. Conversely, tubacin, a specific inhibitor of HDAC-6, does not affect TP expression and slightly reduces TS expression.

**Figure 2:**
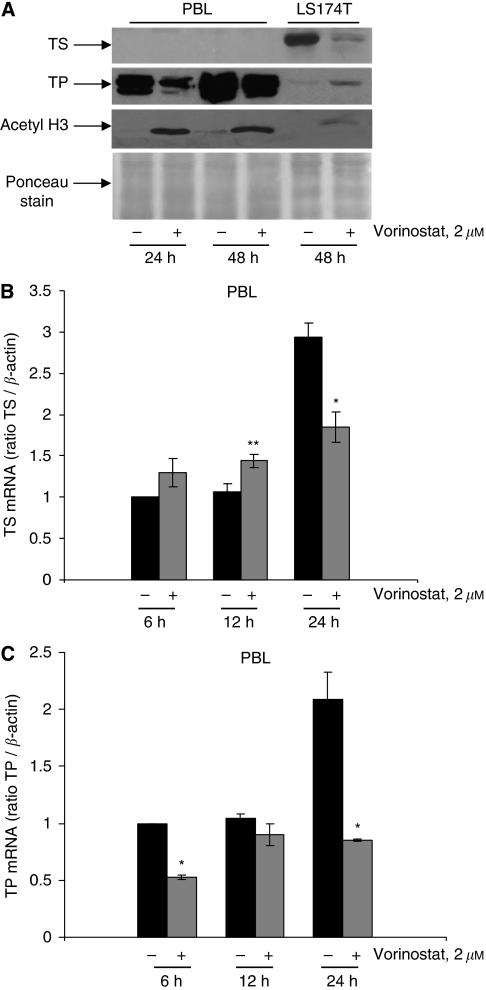
Effect of vorinostat on TS and TP expression in PBLs. (**A**) TS and TP proteins expression were determined by western blotting in PBLs obtained from healthy donor untreated or treated *ex vivo* with vorinostat for 24 and 48 h and compared with LS174T cells. Acetyl histone H3 was used to confirm the effect of vorinostat on PBLs. Ponceau stain was used as protein loading control. (**B**) TS and (**C**) TP mRNA expression were determined by real-time PCR in PBLs untreated or treated with vorinostat at indicated concentrations and harvested at indicated times. Bars, s.d. ^*^P⩽0.005; ^**^P<0.01.

Interestingly, in PBLs from healthy donors treated *ex vivo* with vorinostat, we did not observe TP induction, but rather downregulation of both TP protein (Figure 2A) and mRNA (Figure 2C) expression (2.5-fold peak reduction of TP transcript after 24 h). Moreover, when comparing PBLs with CRC LS174T cells, we observed an undetectable basal level of TS protein unmodified by vorinostat, whereas the TS transcript was slightly modulated by the treatment (1.5-fold reduction after 24 h) (Figure 2A and B). To confirm that PBLs were effectively targeted by vorinostat, a western blot analysis of acetylated histone H3 was performed, demonstrating an increased histone-H3 acetylation at 24 and 48 h of treatment with vorinostat (Figure 2A).

These results suggest that vorinostat may increase sensitivity to fluoropyrimidines such as 5′-DFUR and capecitabine by specifically modulating both TS and TP expression in cancer cells, but not in normal controls.

### *In vitro* synergistic antitumour effects of vorinostat in combination with 5′-DFUR

We next investigated the antitumour effect of vorinostat in combination with the capecitabine metabolite 5′-DFUR. We demonstrated that combined treatment with equipotent doses of vorinostat and 5′-DFUR for 96 h resulted in synergistic antiproliferative effects in LoVo, LS174T and SW620 cells as shown by CIs values that were always lower than 0.8, calculated at 50% (CI_50_) or 75% (CI_75_) of cell lethality (Table 2). Moreover, in all cell lines, we measured a dose reduction in the IC_50_ values (DRI_50_) from 1.4- to 4.4-fold for both vorinostat and 5′-DFUR in combination compared with the concentrations of the two drugs alone.

**Figure 3:**
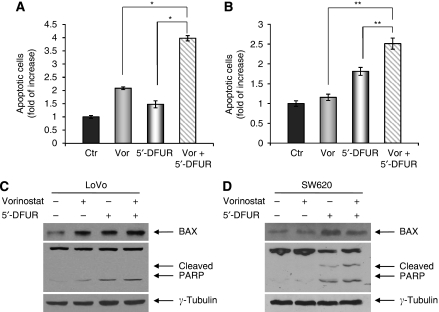
Apoptotic effect of vorinostat in combination with 5′-DFUR on CRC cells. Apoptosis was evaluated by flow cytometry analysis after nuclear DNA staining with propidium iodide in LoVo (**A**) and SW620 (**B**) cells untreated or treated for 72 h with vorinostat (at 0.35 and 0.25 *μ*M, respectively) and/or 5′-DFUR (at 7 and 12 *μ*M, respectively). Bars, s.d. ^*^P⩽0.005. ^**^P<0.05. Western blot analysis of BAX and PARP were performed on LoVo (**C**) and SW620 (**D**) cells untreated or treated for 72 h with vorinostat (0.35 and 0.25 *μ*M, respectively) and/or 5′-DFUR (7 and 12 *μ*M, respectively). *γ*-Tubulin was used as protein loading control.

We next assessed the ability of vorinostat, 5′-DFUR, and their combination to induce apoptosis. In both LoVo and SW620 cells, using low doses (IC_30_^96 h^) of vorinostat (0.35 and 0.25 *μ*M, respectively) and of 5′-DFUR (7 *μ*M and 12 *μ*M, respectively), we observed a significant increase in apoptosis induced by the combination therapy compared with single agent treatments after 72 h exposure (Figure 3A and B).

In the same conditions, in both LoVo and SW620 cells, vorinostat and 5′-DFUR induced the expression of the proapoptotic protein BAX or the cleavage of PARP. These effects were even slightly increased or confirmed by combination treatment (Figure 3C and D). Notably, the low doses of vorinostat employed in the latter experiments were able to significantly modulate TP expression (see Figure 1), suggesting that the enhanced lethality of the combination treatment could be attributed, at least in part, to this mechanism.

### Knockdown of TP reduces the apoptotic effect of vorinostat in combination with 5′-DFUR

To determine if the vorinostat-induced expression of TP is mechanistically correlated with the observed synergism we next evaluated whether TP knockdown by a specific siRNA would affect vorinostat/5′-DFUR-mediated apoptosis.

**Figure 4:**
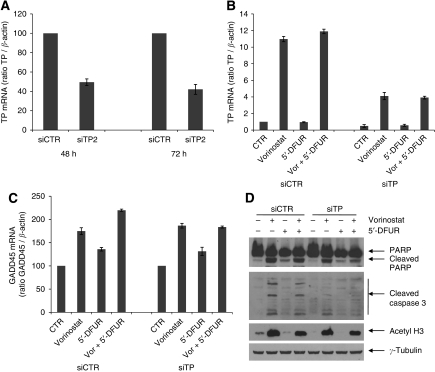
Knockdown of TP reduces the apoptotic effect of vorinostat in combination with 5′-DFUR on SW620 cells. (**A**) Small interfering RNA (siRNA) for TP (siTP) significantly reduced TP mRNA expression determined by real-time PCR both after 48 and 72 h from transfection compared with control siRNA cells (siCTR). siCTR and siTP SW620 cells, 24 h after transfection were untreated or treated for 24 h with vorinostat (2 *μ*M) and/or 5′-DFUR (150 *μ*M) and TP (**B**) and GADD45 (**C**) mRNA expression were determined by real-time PCR. (**D**) Western blot analysis of PARP, cleaved caspase 3 and acetyl histone H3 were performed on siCTR and siTP SW620 cells treated as in **B** and **C**. *γ*-Tubulin was used as protein loading control.

As reported in Figure 4, SW620 cells transfected with siTP construct showed a clear reduction of TP mRNA levels compared with control siRNA transfected cells after 48 and 72 h from transfection (Figure 4A). Moreover, in those cells in which TP had been knocked down the capacity of vorinostat to increase its expression is dramatically reduced compared with control cells (Figure 4B). Notably, in TP knocked down cells we did not observe the increased induction of GADD45, a growth-arrest and DNA-damage-inducible gene, observed in control siRNA-transfected cells upon vorinostat/5′-DFUR combination treatment compared with single agent treatments (Figure 4C). Similarly both PARP and caspase 3 cleavage induced by vorinostat/5′-DFUR combination treatment were significantly reduced in TP knocked down cells (Figure 4D). Altogether these findings confirmed that at least in part, the upregulation of TP expression induced by vorinostat is critical for the synergistic antiproliferative and apoptotic cell death induced by vorinostat/5′-DFUR combination.

### *In vivo* synergistic antitumour effect of vorinostat in combination with capecitabine

**Figure 5:**
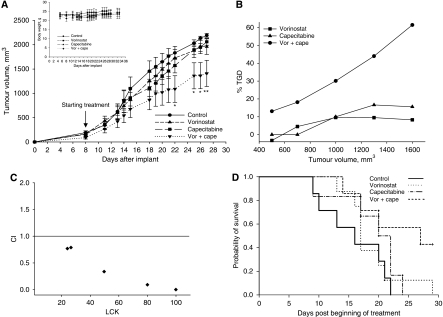
Antitumour activity of vorinostat and capecitabine on established colorectal cancer xenografts. SW620 cells (5 × 10^6^) were subcutaneously injected into athymic mice as described in the Materials and Methods. When established tumours were palpable, mice were treated with vorinostat (100 mg kg^−1^ p.o.), capecitabine (359 mg kg^−1^ p.o.) or both drugs 5 days/week for two weeks. (**A**) Mean±s.d. tumour volume measured at pre-specified time points (*n*=9). ^*^P⩽0.001; and ^**^P⩽0.01. Inset, body weight measured three times per week. (**B**) Tumour growth delay (TGD), determined as %TGD=((T – C)/C) × 100, where *T* and *C* are the mean times expressed in days for the treated or control groups, respectively, to reach a defined tumour volume (see Materials and Methods). (**C**) *In vivo* vorinostat plus capecitabine combination studies evaluated by CalcuSyn. For the calculation of CI, the values of log cell kill (LCK) for a fixed tumour volume were considered (see Materials and Methods). (**D**) Effect of vorinostat and/or capecitabine on the survival of SW620 xenograft mice. Survival was analysed by Kaplan–Meier survival curves (*P*=0.03 combination *vs* control). Mice were killed by cervical dislocation when evidence of advanced bulky disease was present (cutoff, mean tumour volume=2000 mm^3^), and the day of killing was considered the day of death for survival evaluation.

To determine *in vivo* the presence of the synergistic antitumour effects that were demonstrated *in vitro*, we evaluated vorinostat in combination with capecitabine in a SW620 cell xenograft experimental model in athymic nude mice by measuring tumour volume (Figure 5A), TGD (Figure 5B), CIs (Figure 5C) and survival (Figure 5D). A total of 36 xenografted mice were randomly assigned to receive sub-therapeutic doses of vorinostat (100 mg kg^−1^ p.o.), capecitabine (359 mg kg^−1^ p.o.), both drugs in combination, or their vehicles as a control. Treatments were administered 5 days/week for two weeks.

At day 27, which represents the median survival duration of mice in the control group, the combination treatment induced a significant inhibition (36±7%, *P*<0.005) of tumour growth compared with single agent treatments (Figure 5A). The resulting TGD reached a peak of >60%, indicating that the rate of tumour growth in the control, at that point, was almost 2.4-fold higher than in the combination treatment setting (Figure 5B). Single-agent treatment of the indicated doses resulted in a slight growth inhibition with no significant effects observed on TGD.

Furthermore, synergistic effects between vorinostat and capecitabine were also confirmed by the evaluation of CIs reported *vs* LCK (see Materials and Methods; Figure 5C). As a consequence, substantial increases in survival were observed only in the group treated with vorinostat plus capecitabine, as four out of nine mice were still alive 30 days post-implantation (*P*<0.05).

Combinatory treatment of vorinostat plus capecitabine was well tolerated, as shown by the maintenance of body weight (inset in Figure 5A) and the absence of other signs of acute or delayed toxicity.

### Vorinostat/capecitabine treatment decreased proliferation markers, induced apoptosis and modulated TS and TP expression in tumour xenografts

**Figure 6:**
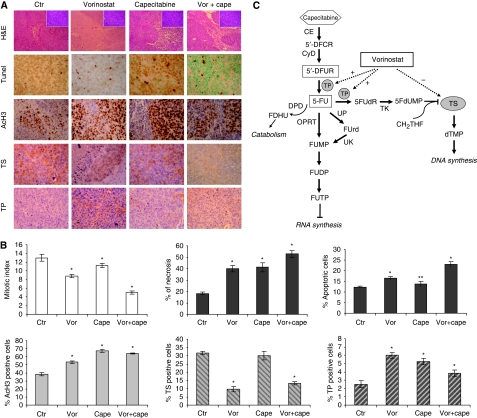
Effect of vorinostat/capecitabine treatment on proliferation, apoptosis, acetylation of histone H3, and expression of TS and TP in SW620 xenograft tumours. (**A**) Paraffin-embedded tissue was generated from each tumour for hematoxylin and eosin stain (H&E), TUNEL assay and immunohistochemistry analysis for the indicated markers as described in the Materials and Methods. Images were captured with a 20 × or 40 × objective on a light microscope. (**B**) Necrotic area was evaluated as the percentage of necrosis inner to tumoural nodule. Mitosis was evaluated as number of mitotic figures in 10 high-magnification fields (Inset in A). Tumour sections stained for TUNEL, TS, TP or acetil-H3 were scored semiquantitatively for the percentage of positive cells. The group means were calculated for *n*=5 to seven tumours per group. All of the data are presented as the average±s.d. ^*^P⩽0.001; and ^**^P⩽0.05, statistical significance compared with control. (**C**) Schematic depicting the mechanism of antitumour activity of capecitabine and the effects of vorinostat. 5-FUTP=5-fluorouridine triphosphate; 5FdUTP=5-fluorodeoxyuridine triphosphate; 5FdUMP=5-fluorodeoxyuridine monophosphate; CE=carboxylesterase; CyD=cytidine deaminase; FdUrd=2′-deoxy-5-fluorouridine; FdUMP=5-fluorodeoxyuridine monophosphate; OPRT=orotate phosphoribosyltransferase; TK=thymidine kinase; TP=thymidine phosphorylase; TS=thymidylate synthase.

Analysis of mitotic and necrotic cells on H&E stained slides from SW620 xenograft tumours demonstrated that combination treatments induced a significant reduction of mitotic cells and a contextual increase in the percentage of necrotic cells compared with the untreated group or single-agent treatment group. Moreover, a significant increase of apoptosis as demonstrated by TUNEL assay was observed in the vorinostat plus capecitabine group compared with the untreated group or single-agent treatment group (Figure 6A and 6B). Notably, as shown by IHC analysis of xenograft tumours, the downregulation of TS and the upregulation TP were also confirmed *in vivo* in the vorinostat and the vorinostat plus capecitabine groups. Interestingly, TP protein expression was also increased by capecitabine alone compared with untreated controls. Finally, analysis of histone-H3 acetylation demonstrated a significant increase in both the vorinostat and vorinostat plus capecitabine groups compared with controls, as expected, but we also observed an evident increase in the capecitabine group.

Taken together these data confirmed our *in vitro* findings that modulation of both TS and TP by vorinostat may be a potential mechanism for the synergistic antitumour interaction observed between vorinostat and capecitabine.

## Discussion

The results of the present study suggest a benefit from combining vorinostat with capecitabine in CRC, indicating that the modulation of TS and TP expression by vorinostat may explain the synergistic interaction between these two drugs observed both *in vitro* and *in vivo*. We demonstrated that coadministration of these agents might represent a worthy strategy for more effectively targeting *de novo* synthesis of dTMP and subsequent DNA synthesis by fluoropyrimidines. Indeed, we showed that the antiproliferative effect induced by vorinostat in CRC cells and in xenograft tumours was paralleled by downregulation of TS protein, the crucial enzyme for thymidilate synthesis and the target of 5-FU, and by upregulation of TP protein, a critical enzyme in the final step of the metabolic transformation of capecitabine to 5-FU. Thymidine phosphorylase, by reversibly converting 5-FU to 5′-FUDR, which can be subsequently converted to 5FdUMP, may also favour the formation of the stable inactive ternary complex between this latter 5-FU-metabolite, the TS enzyme and the methyl donor CH2THF (Figure 6C). Consequently, we demonstrated that simultaneous exposure of vorinostat and the capecitabine metabolite 5′-DFUR *in vitro* resulted in synergistic antiproliferative and proapoptotic effects in all CRC cell lines examined, independent of p53 status and including cells strongly resistant to 5′-DFUR.

The synergistic effect demonstrated *in vitro* was also confirmed *in vivo* in a human CRC xenograft model, in which a marked inhibition of tumour growth, increased apoptosis and increased survival were observed in vorinostat plus capecitabine combination compared with single agent treatments.

The association of treatment with pharmacodynamic readout of efficacy represents a major challenge for novel targeted drugs. Notably, our data demonstrated that the antitumour activity we observed *in vivo* correlated with the induction of histone acetylation, a marker of HDAC-Is activity, as well as with a reduction in the markers of proliferation and induction of apoptosis on tumour xenografts. Moreover, the modulation of both TS and TP expression by vorinostat observed *in vitro* was clearly confirmed *in vivo*, providing additional insights into the mechanism of the synergistic interaction between vorinostat and capecitabine. The observation that vorinostat downregulates TS protein expression both *in vitro* and *in vivo* as a consequence of vorinostat-induced attenuation of TS mRNA transcript confirmed previous findings from our group and from others (Glaser *et al*, 2003; Lee *et al*, 2006; Tumber *et al*, 2007; Di Gennaro *et al*, 2009; Fazzone *et al*, 2009). Thymidylate synthase protein was not detectable by western blot analysis in nontumoural PBLs, and vorinostat induced a late and less pronounced inhibition of TS transcription in these cells compared with CRC cells. On the other hand, to our knowledge, this is the first study to show that vorinostat modulates the expression of TP both *in vitro* and *in vivo.* Significantly, this effect was also shared by other pan-HDAC-Is such as LBH589 or TSA and by class I HDAC-Is such as VPA and MS275, but not by the specific inhibitor of HDAC-6 tubacin, indicating that class I HDAC targeting is crucial for TP induction.

This is also the first study to demonstrate synergistic antitumour effects between vorinostat and capecitabine *in vivo* in a preclinical model. A recent paper by Guarcello *et al* (2008) showed that TP expression can be suppressed in human cancer cells by promoter methylation. This paper showed that treatment with methylation-inhibitor 5-aza-2′-deoxycytidine alone or in combination with HDAC-I TSA resulted in increase TP mRNA and protein levels. We showed that the upregulation of TP in CRC cells and in human cancer cells of different tissues of origin (unpublished observation) was independent of the basal level of TP. Notably, sublethal doses of vorinostat, far below the maximum serum concentrations reached with single agent therapy or in combination treatment in cancer patients, were able to significantly modulate TP expression within 24 h, suggesting that the enhanced lethality of the combination treatment could be primarily, if not exclusively, attributed to this mechanism. In this regard, we also showed that TP knockdown by a specific siRNA significantly impairs the synergistic apoptotic cell death induced by vorinostat/5′-DFUR combination.

Intrinsic or acquired resistance to fluoropyrimidines is often associated with TS overexpression, and it has also correlated with low or deficient intratumoural TP activity (Salonga *et al*, 2000; de Bruin *et al*, 2003). For this reason, our findings are clinically relevant. Indeed, in this study, the induction of TP by vorinostat and the synergistic interaction of vorinostat with 5′-DFUR or capectabine is also evident in two cell lines such as LS174T or SW620, both of which express undetectable levels of TP protein and very low levels of TP mRNA, as well as both being strongly resistant to 5′-DFUR. Notably, in nontumoural PBLs treated *ex vivo* by vorinostat, we observed a reduction of both TP protein and mRNA expression. Consequently, we can assume that as demonstrated for other genes, TP can be specifically modulated by HDAC-Is in tumour cells in which it is deregulated either by promoter methylation or other mechanisms, whereas normal cells are not affected. This latter observation suggests that the increased antitumour effect observed in tumour cells both *in vitro* and *in vivo* by combining vorinostat with fluoropyrimidines should not translate into an increased toxicity in normal cells. Indeed, no additional toxic effect was observed *in vivo* in the combination treatment setting compared with single-drug treatment. We are currently evaluating in-depth the mechanism as well as the functional significance of the selective induction of TP in tumour cells.

We are aware that TP showed a strong sequence homology to the pro-angiogenic platelet derived endothelial cell growth factor (PD-ECGF), such that the two enzymes are considered identical (Ishikawa *et al*, 1989; Usuki *et al*, 1992; Moghaddam *et al*, 1995). It has been shown that PD-ECGF/TP may contribute to angiogenesis, tumour progression and metastasis via mechanisms that remain to be defined (reviewed in Liekens *et al*, 2007; Koopman *et al*, 2009). We have not investigated the effect of vorinostat on signal transduction pathways leading to angiogenesis and/or metastasis in our preclinical models; however, several reports have clearly shown that HDAC-Is, including vorinostat, block tumour invasion and metastasis as well as tumour-induced angiogenesis (Kim *et al*, 2004; Qian *et al*, 2006; Shankar *et al*, 2009).

Capecitabine was designed to take advantage of the increased levels of TP observed in tumours as opposed to normal tissues, potentially allowing for selective toxicity in tumours (Bollag and Hartmann, 1980; Walko and Lindley, 2005), and is a valuable substitute for a bolus or infusion of 5-FU either as monotherapy or in combination with other cytotoxic drugs in the treatment of cancer types for which it is currently approved, such as breast and CRCs. However, strategies for the potentiation of anticancer activity of capecitabine are required to improve the therapeutic index of this drug, and several current clinical efforts are focused on the evaluation of TP-inducible therapy in combination with capecitabine for cancer treatment (Liekens *et al*, 2007). Although there is no direct demonstration of the role of TP, the clinical investigation of TP-inducible chemotherapeutics, such as taxanes, cisplatin or cyclophosphamide, in combination with capecitabine showed increased response rate, time to progression and survival in breast cancer patients in phase III studies. Promising results were also seen in gastric and NSCLC in phase II studies (Kindwall-Keller *et al*, 2005; Walko and Lindley, 2005; Ajani, 2006; Gelmon *et al*, 2006).

Recently, two phase I pharmacokinetic and pharmacodynamic studies have defined the maximum tolerated dose of vorinostat in combination with FOLFOX (FOLFOX, fixed dose of 5-Fluorouracil, leucovorin and oxaliplatin) or 5-FU/LV chemotherapy regimens (Fakih *et al*, 2009, 2010) in CRC. However, no consistent effect of vorinostat on TS expression in patient tumour samples was demonstrated in both studies. Interestingly, clinical activity was noted also in patients with 5-FU-refractory CRC (Fakih *et al*, 2010), which agrees with our previous data demonstrating that vorinostat sensitises 5-FU-resistant CRC cells to 5-FU. In conclusion, although other enzymes such as dihydropyrimidine dehydrogenase, which regulate the rate limiting step in the catabolism of 5-FU as well as the disregulation in tumour cells of critical pathways regulating survival, growth arrest or apoptosis, could be related to the antitumour efficacy of fluoropyrimidines (Ishikawa *et al*, 1999; Yen and McLeod, 2007) our results indicate that vorinostat has the unique capability to modulate not only TS but also TP expression in tumour cells (Figure 6C) and, consequently, can synergise with capecitabine. In addition, both vorinostat and capecitabine are drugs that can be administered orally with consequent increased compliance for the patients. In conclusion, our study showed that the combination of vorinostat and capecitabine is a feasible and promising chemotherapeutic strategy for colon cancer treatment and should be clinically explored.

## Figures and Tables

**Table 1:**
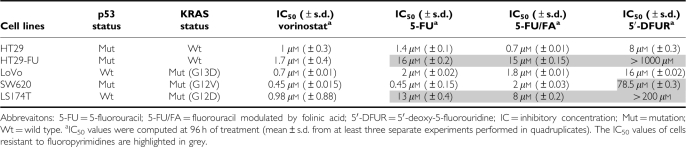
Sensitivity of colorectal cancer cell lines to vorinostat and fluoropyrimidines (5-FU, 5-FU/FA, 5′-DFUR)

**Table 2:** Combination index (CI) and dose reduction index (DRI) values for vorinostat and 5′-deoxy-5-fluorouridine (5′-DFUR) combination treatment

			**DRI at IC_50_ ±s.d.**	
**Cell lines**	**CI_50_ ±s.d. vorinostat + 5′-DFUR^a^**	**CI_75_ ±s.d vorinostat + 5′-DFUR^a^**	**Vorinostat**	**5′-DFUR^b^**
LoVo	0.78±0.13	0.76±0.1	2.3±1	4.4±2
LS174T	0.69±0.09	0.6±0.2	1.4±0.7	2±0.5
SW620	0.76±0.1	0.57±0.23	3.1±0.8	2.3±0.2

a CI values (mean±s.d. from at least three separate experiments performed in quadruplicates) computed at 50 and 75% of cell kill (CI_50_ and CI_75_, respectively) according by CalcuSyn software after 96 h of treatment. Combinations were considered strongly synergistic when CIs were below 0.9.

b DRI values (mean±s.d. from at least three separate experiments performed in quadruplicates) represents the order of magnitude (fold) of dose reduction obtained for IC_50_ (DRI_50_) in combination setting compared with each drug alone.
